# The Effects of Individual Components of E-Cigarettes on Ion Transport and Airway Surface Liquid Height in Human Bronchial Epithelial Cells

**DOI:** 10.3390/medicina61030526

**Published:** 2025-03-17

**Authors:** Ozge Beyazcicek, Robert Tarran, Recep Ozmerdivenli, Ersin Beyazcicek

**Affiliations:** 1 Department of Physiology, Faculty of Medicine, Duzce University, 81620 Duzce, Turkey; 2 Division of Genetic, Environmental and Inhalational Disease, Department of Internal Medicine, Kansas University Medical Center, Kansas City, KS 66103, USA; 3 Department of Cell Biology and Physiology, The University of North Carolina at Chapel Hill, Chapel Hill, NC 27599, USA; 4 Department of Physiology, Faculty of Medicine, Aydin Adnan Menderes University, 09010 Aydın, Turkey

**Keywords:** JUUL, short-circuit current, transepithelial resistance, cytotoxicity

## Abstract

Background and Objectives: The rising popularity of new-generation electronic cigarettes (e-cig) like JUUL necessitates a better understanding of their impact on respiratory and other body systems, as the effects of JUUL’s components remain unclear. This study aimed to investigate the effects of JUUL components on ion channels and airway surface liquid (ASL) height in human bronchial epithelial cells (HBECs). Furthermore, the cytotoxic effects of these components were investigated in human embryonic kidney 293T (HEK293T) cells. Materials and Methods: The components tested included nicotine salt (NicSalt), benzoic acid (BA), sodium hydrogen tartrate (NaTar), propylene glycol/vegetable glycerin (PG/VG), freebase nicotine (FBNic) and nicotine salt+benzoic acid (NicSalt+BA). Each component was prepared at 100 µM, and HBECs were exposed for 24 h to measure ASL height, short-circuit current (*I_sc_*), and transepithelial electrical resistance (TEER). Results: Initial exposure (0 h) to these substances did not significantly alter ASL height. However, after 2 h, FBNic-treated HBECs exhibited a significant reduction in ASL height compared to NicSalt and other tested substances, with the most pronounced decrease observed at the 6th hour. This effect persisted over prolonged exposure, suggesting a cumulative impact on airway hydration and epithelial function. Additionally, adenosine administration did not induce a significant increase in ASL height. NicSalt, BA, and FBNic were found to disrupt ion balance in HBECs, affecting ion channels and ASL homeostasis while significantly decreasing TEER. In terms of cytotoxicity, NicSalt, and benzoic acid demonstrated minimal cytotoxicity at low concentrations, whereas FBNic showed significantly higher cytotoxicity at moderate levels. Conclusions: In conclusion, this study highlights that e-cigarette components can disrupt airway surface liquid homeostasis by affecting ion channel activity, compromise epithelial barrier integrity by reducing transepithelial electrical resistance, and emphasize the importance of their cytotoxic effects.

## 1. Introduction

The discovery and use of nicotine by humans date back to centuries. Since its discovery, the quest to use nicotine in different ways has been rapidly increasing with the development of technology. As the main form of nicotine consumption, users generally prefer tobacco cigarettes. In addition, nicotine gum, nicotine patches, and electronic cigarettes, which are increasingly used today, are among the other products that provide nicotine to users besides tobacco cigarettes.

The advent of electronic nicotine delivery system (ENDS) devices, often marketed as safer alternatives to traditional tobacco smoking, has significantly reshaped the dynamics of smoking and nicotine consumption.

Today, health authorities in some countries recommend e-cigarettes for those who want to reduce or quit smoking [1]. However, the rapid evolution of e-cigarette technology, with advanced features and diverse nicotine formulations, requires thorough investigation into its health impacts. A key concern is how e-cigarette vapor exposure affects the respiratory system, particularly its effects on ion channels and transporters essential for lung function.

JUUL is a pod-based electronic cigarette with pre-filled cartridges containing nicotine salt (61.6 ± 1.5 mg/mL in a 0.7 mL solution), flavorings, and benzoic acid (44.8 ± 0.6 mg/mL). Propylene glycol (PG) and vegetable glycerin (VG) form the bulk of the liquid, suspend nicotine and flavors, and facilitate aerosolization. Benzoic acid is a compound naturally present in tobacco [2]. As of August 2018, JUUL has launched cartridges with two different nicotine concentrations, 5% (59 mg/mL) and 3% (35 mg/mL). Each cartridge is equivalent to one pack of cigarettes (approximately 200 puffs) and is available in eight different flavor options: mango, mixed fruit, crème brûlée, mint, Virginia tobacco, classic tobacco, menthol, and cucumber [2].

Although the mechanisms of nicotine delivery differ, both traditional cigarettes and e-cigarettes aim to provide effective nicotine absorption through varying chemical formulations. Freebase nicotine, the most common form in e-liquids, is unprotonated and rapidly absorbed through biological membranes due to its alkaline nature. However, its volatility causes rapid deposition in the upper respiratory tract, leading to harsh inhalation, irritation, dryness, and inflammation [3]. Nicotine salts, the protonated form of nicotine found in tobacco, reduce harshness during vaping and allow deeper lung penetration. Compared to free-base nicotine, nicotine salts lead to higher plasma nicotine concentrations. This form also minimizes throat irritation, encourages deeper inhalation, and increases nicotine delivery to the alveoli [4].

E-liquids primarily affect airway surface liquids in the respiratory system. The respiratory tract is lined with a thin periciliary layer and a layer of mucus covering it, which is called airway surface fluid or liquid (ASL), which plays an important role in maintaining the sterility of the lung [5]. In a normal respiratory tract, the mean height of the airway surface liquid layer is 7 µm. The primary ions affecting airway surface liquid volume and mucus clearance—Cl^−^, Na^+^, and water—can be altered by e-liquid contents through ion exchanges, changing liquid height and thickness, and making the lungs more vulnerable to infections [6].

Key ion channels influencing ASL volume and mucus clearance include epithelial sodium channels (ENaC), cystic fibrosis transmembrane regulator (CFTR), and calcium-activated chloride channels (CaCC). ENaC transports Na^+^ ions across the epithelial cell membrane [7], while CFTR regulates Cl^−^ transport and Na^+^ entry [8]. CaCC channels, activated by increased intracellular Ca^+2^, also regulate Cl^−^ secretion [9,10]. The interaction between CFTR, CaCC, and ENaC controls the airway mucosal layer, with CFTR maintaining basal mucus levels and CaCC acting as an acute regulator of airway surface fluid.

As a next-generation e-cigarette, JUUL’s impact on the respiratory system, ion channels, and cytotoxicity must be examined [11]. While previous studies have examined the effects of e-cigarette aerosols and nicotine on airway epithelial function, our study investigates the impact of individual JUUL components on ASL height, ion transport mechanisms, and epithelial barrier integrity. This targeted approach allows us to distinguish the specific contributions of each component, providing a more precise understanding of their physiological effects. By focusing on individual substances, we contribute novel insights into how different JUUL components independently modulate airway hydration and epithelial function, which has not been previously addressed in detail in the literature (Table 1). This study aims to investigate the effects of JUUL components, a next-generation e-cigarette, on airway surface liquid height in human bronchial epithelial cells (HBECs), to assess their effects on ENaC, CFTR, and CaCC channels through short-circuit current measurements, to evaluate barrier integrity using transepithelial electrical resistance measurements, and to reveal their cytotoxic effects compared to freebase nicotine.

## 2. Materials and Methods

### 2.1. Chemicals and Reagents

Phosphate buffer saline (PBS) (Thermo Fisher, Gibco, Waltham, MA, USA), nicotine salt (nicotine hydrogen tartrate salt, Sigma #SML1236), benzoic acid (Sigma #242381), sodium hydrogen tartar (Sigma #71995), propylene glycerol, vegetable glycerin (with the ratio of 55/45%), and freebase nicotine (Sigma Aldrich, St. Louis, MO, USA), and dimethyl sulfoxide (DMSO) (Sigma #D2650) were used in this study. Reagents for cell culture were purchased from Gibco (Waltham, MA, USA).

### 2.2. Cell Cultıre

Primary human bronchial epithelial cells (HBECs) closely mimicking in vivo airway epithelial morphology were used to investigate airway physiology. Human embryonic kidney 293T (HEK293T) cells, an immortalized human embryonic kidney cell line, were also employed to assess cytotoxicity.

### 2.3. Cell Culture for HBECs

Human lung primary bronchial epithelial cells (HBECs) were obtained from the University of North Carolina at Chapel Hill, Marsico Lung Institute CF Center Tissue Core, under protocols approved by the UNC Institutional Committee for the Protection of the Rights of Human Subjects and with ethical approval from the UNC Biomedical Review Board (protocol #03-1396). HBECs were seeded onto a 24-well plate by using transparent transwells (Oxyphen AG Giessereistrasse, Wetzikon, Switzerland), with a diameter of 6.5 mm and a pore opening of 0.4 µm, coated with collagen from human placenta (Sigma Aldrich, St. Louis, MO, USA) at a density of 0.5 × 10^5^ cells per transwell to measure ASL height. For short-circuit current and transepithelial resistance measurements, HBECs were seeded onto a 12-well plate by using transparent transwells (Corning Costar, cat no:3460, Corning, NY, USA), with a diameter of 12 mm and a pore opening of 0.4 µm, coated with collagen from human placenta (Sigma Aldrich, St. Louis, MO, USA) at a density of 2 × 10^5^ cells per transwell. The cells were grown under air–liquid surface conditions using the UNC air–liquid (ALI) medium [19], which was added to the serosal side [19]. The cells were incubated (Nuaire, 2100 Fernbrook Lane, Plymouth, MN, USA) for 3–4 weeks in 5% CO_2_ and 95% O_2_ at 37 °C with the apical side facing the air and the basolateral side facing the liquid media to differentiate the cells and reach a ciliated structure. During the incubation period, the media of the transwells were changed every two days and the cells were washed with PBS three times a week to clean them of excess mucus.

### 2.4. Cell Culture for HEK293T

HEK293T cell lines were acquired from ATCC, cultured in Dulbecco’s modified Eagle’s medium (DMEM, high glucose, Gibco), enriched with 10% fetal bovine serum (Corning) and 1x penicillin/streptomycin (Sigma), and incubated at 37 °C with 5% CO_2_. The cells were seeded onto 384-well plates Corning #3764 at 5000 cells per well for live-dead assays.

### 2.5. The ASL Height Measurements

All differentiated HBECs were washed with PBS twice to measure ASL height, and excess mucus aspirated with a Pasteur pipette before starting the experiment. To label the ASL on epithelial cells, 0.1 mg/mL dextran (Life Technologies, D-1816, Eugene, OR, USA) was added at a 100 µM concentration in PBS (Table 2) and applied to transwells (14 µL per transwell) according to substance groups. Since ASL evaporates quickly upon air exposure, 40 µL of perfluorocarbon was added apically to each transwell before confocal imaging (Leica TCS SP5; Leica Microsystems, Wetzlar, Germany) to prevent evaporation. Cultures were placed in Ringer solution on the serosal side and positioned on an inverted confocal microscope stage. ASL height was imaged at 0, 2, 6, and 24 h. After the 24 h measurement, adenosine dissolved in perfluorocarbon was added apically to activate CFTR via A2B adenosine receptors, and ASL height was re-imaged at 0 and 30 min. Between measurements, HBECs were incubated (37 °C/5% CO_2_). ASL height was determined using a Leica SP5 confocal microscope (63× glycerol immersion lens, 1.2–1.3 NA) with XZ scanning at five predetermined points (one central, four peripheral). ImageJ (version 1.53s, NIH Freeware) was used for analysis.

### 2.6. The Ussing Chamber Measurements

After HBECs differentiation, 100 nM dexamethasone, which expresses ENaC channels [20], was added to the culture media, followed by the application of substances (20 µL per culture) (Table 2) apically and incubated (37 °C/5% CO_2_) overnight. Transepithelial electrical resistance (TEER) and short-circuit current (*I_sc_*) were monitored at regular intervals under voltage clamp conditions using the Ussing chamber (VCC MC8 Voltage Current Clamp, Physiologic Instruments, San Diego, CA, USA) [21] to which 3% KBR (glucose-free) agar bridges were connected. The chamber parts of the Ussing Chamber separated by a thin layer, in which the HBECs are placed. Both sides were with Krebs Bicarbonate Ringer (KBR) solution (in mM) 115 NaCl, 2.4 K_2_HPO_4_, 0.4 KH_2_PO_4_, 24 NaHCO_3_, 10 D-glucose, 1.2 CaCl_2_, and 1.2 MgCl_2_ (pH 7.4), constituting a physiological solution, and bubbled with 5% CO_2_/95% O_2_. After equilibration, the cells were placed into the chambers, and the mucosal solution was supplemented with 100 µM amiloride (ENaC inhibitor), 10 µM forskolin (CFTR stimulator), 10 µM CFTRinh-172 (CFTR blocker), and 10 µM UTP (Ca^2+^-activated Cl^−^ channel stimulator). During the recording, all chambers were kept at 37 °C. The *I_sc_* and TEER of the cells were measured. Data were analyzed using Acquire & Analyze software (version 1.2, Physiologic Instruments, San Diego, CA, USA) [22].

### 2.7. Cytotoxicity Tests

The concentrations used in this study were selected based on previously published dose–response studies available in the literature, to facilitate comparison with existing findings [16]. All chemicals were prepared as 105 mM and 35 mM stock solutions and were then serially diluted to treat the HEK293T cells. The doses and duration of exposure to the substances to determine the dose–response shown in Table 2. The cells treated with serial dilutions of the substances were incubated overnight at 37 °C with 5% CO_2_. In addition, to assess the values for 100% dead cells, DMSO was added to certain wells as a positive control. After 24 h, all cells were washed with PBS and stained with 1.0 μM calcein-AM (Corning #354216) and 1.5 μM propidium iodide (Sigma #P4170) for 30 min at 37 °C to distinguish live and dead cells. Dose–response experiments followed previously described protocols [23]. Live-dead staining was imaged using Cytation 5 (BioTek, Winooski, VT, USA), to determine the cytotoxic effects.

Imaging was performed using excitation/emission wavelengths of 469/525 nm for calcein-AM and 531/647 nm for propidium iodide with the BioTek Cytation 5 [23]. Each dose was performed in triplicate (*n* = 3) on three separate occasions (*n* = 3). The obtained live/dead data of the cells exposed to substances were analyzed.

### 2.8. Statistical Analysis

All data sets were first evaluated for normality using the Shapiro–Wilk test. For data sets exhibiting normal distribution, comparisons among multiple groups were performed using one-way analysis of variance (ANOVA). When ANOVA indicated significant differences, we compared pairwise with Tukey’s post hoc test to identify specific group differences. For data sets not normally distributed, the non-parametric Kruskal–Wallis analysis was employed, followed by Dunn’s multiple comparisons test for pairwise group analysis. *p*-values ≤ 0.05 were considered significant. Experiments were repeated at least three separate times.

## 3. Results

### 3.1. The Effects of the JUUL Components on the ASL Height

**Figure 1:**
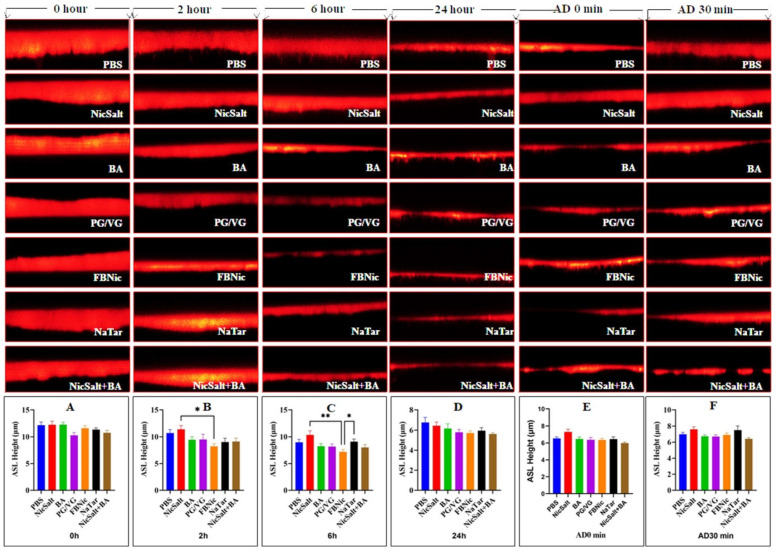
Representative XZ-confocal micrographs of ASL height (**red**) of HBECs after 0–24 h exposure to e-cig components or PBS. (**A**) Bar graph of ASL heights at 0 h after exposure to e-cig components or PBS, (**B**) bar graph of ASL heights at 2 h after exposure to e-cig components or PBS, (**C**) bar graph of ASL heights at 6 h after exposure to e-cig components or PBS, (**D**) bar graph of ASL heights at 24 h after exposure to e-cig components or PBS, (**E**) bar graph of ASL heights at 0 min after adenosine exposure, and (**F**) bar graph of ASL heights at 30 min after adenosine exposure (* *p* < 0.05 and ** *p* < 0.01).

In order to maintain effective clearance and adequate hydration of mucus, normal airway epithelia must preserve the volume and height of ASL through ion transport. To determine the effect of PBS, NicSalt, BA, PG/VG, FBNic, and NaTar on the ASL height of HBECs, measurements were taken using XZ confocal microscopy (Figure 1).

Measurements were taken from three different regions of each ASL image. There was a wide range in ASL heights in this image set, from very narrow to quite wide (Figure 1).

To image ASL height accurately, each culture was washed apically with PBS before substances mixed with dextran were added to the cultures. Directly following the application of the dye with substances, at 0 h, mean ASL height was found to be (in µm) for PBS ~12.18, NicSalt ~12.25, BA ~12.28, PG/VG ~10.28, FBNic ~11.62, NaTar ~11.35 and NicSalt+BA ~10.76. At this time point, mean ASL height was not significantly different between the HBECs cultures treated with the substances (*p* = 0.07) (Figure 1A). The mean ASL height of the HBECs with substances after 2 h was (in µm) for PBS ~10.72, NicSalt ~11.41, BA ~9.44, PG/VG ~9.48, FBNic ~8.21, NaTar ~9.03 and NicSalt+BA ~9.11. Analysis of the data obtained in this manner showed a significant difference in mean ASL height between NicSalt and FBNic (*p* = 0.03) (Figure 1B). The mean ASL height of the FBNic was lower than that of the NicSalt. The mean ASL heights obtained at the 6th hour were as follows (in µm) for PBS ~8.97, NicSalt ~10.36, BA ~8.24, PG/VG ~8.17, FBNic ~7.20, NaTar ~9.08 and NicSalt+BA ~8.01 (Figure 1C). Based on the analysis of the collected data, the mean ASL height differences between FBNic, NicSalt, and NaTar were statistically significant, and FBNic were observed to have a lower mean ASL height compared to both NicSalt and NaTar (*p* = 0.006 and *p* = 0.04, respectively). The obtained mean ASL height from the images of the cultures at 24 h determined as (in µm) for PBS ~6.77, NicSalt ~6.44, BA ~6.16, PG/VG ~5.79, FBNic ~5.70, NaTar ~5.92 and NicSalt+BA ~5.61 and there was no significant difference in the mean ASL height between the HBECs cultures treated with the substances (*p* = 0.27) (Figure 1D). Next, these HBECs were exposed to adenosine, which triggered ASL release [24]. Mean ASL heights (in µm) were measured immediately after adenosine addition (0 min) as follows: PBS ~6.52, NicSalt ~7.31, BA ~6.46, PG/VG ~6.36, FBNic ~6.34, NaTar ~6.44, and NicSalt+BA ~5.97. At 30 min, the values were: PBS ~6.99, NicSalt ~7.60, BA ~6.73, PG/VG ~6.69, FBNic ~6.90, NaTar ~7.51, and NicSalt+BA ~6.42 (Figure 1E). No significant difference was observed between time points (*p* = 0.057, *p* = 0.15, respectively)

### 3.2. The Effects of the JUUL Components on the Ion Transportation

**Figure 2:**
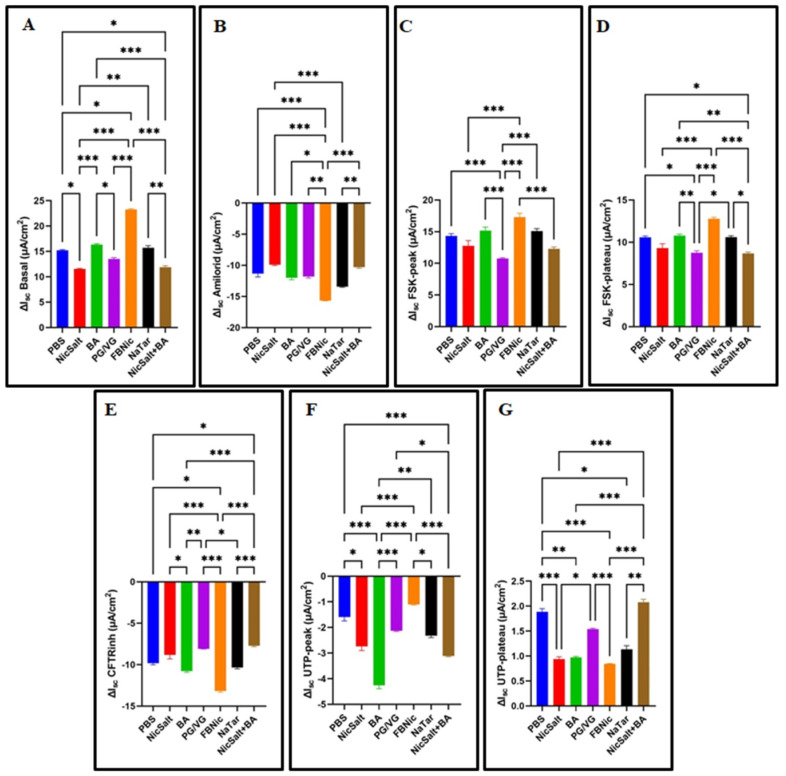
Short-circuit current (*I_sc_*) changes in HBECs after 24 h of exposure to e-cig components or PBS. Changes in short-circuit current at baseline (**A**) and upon the addition of amiloride (**B**), forskolin with FSK-peak (**C**) and FSK-plateau (**D**), CFTRinh-172 (**E**), and the addition of UTP with UTP-peak (**F**) and UTP-plateau (**G**) were measured (* *p* < 0.05, ** *p* < 0.01 and ***p < 0.001).

To investigate the effects of 24 h exposure to e-cigarette components on ion transport in HBECs, Δ*I_sc_* values were measured using Ussing chambers equipped with Ag/AgCl electrodes. Following exposure, basal activity and the responses of ENaC, CFTR, and CaCC channels to specific modulators (amiloride, forskolin, CFTRinh-172, and UTP) were analyzed. The results revealed statistically significant differences in Δ*I_sc_* among different exposure groups (*p* < 0.001) (Figure 2). Notably, NicSalt exposure led to a significant decrease in basal Δ*I_sc_* (Figure 2A). FBNic exposure resulted in a more significant reduction in amiloride-inhibited ENaC Δ*I_sc_* (Figure 2B) and CFTRinh-172 Δ*I_sc_* (Figure 2E) compared to other substances. Additionally, forskolin-activated CFTR responses were attenuated by PG/VG exposure, showing the lowest Fsk*I_sc_*-peak and Fsk*I_sc_*-plateau values (Figure 2C,D). UTP-activated CaCC channel recordings indicated that BA exposure led to the lowest Δ*I_sc_* at UTP*I_sc_*-peak, while FBNic exposure caused the lowest Δ*I_sc_* at UTP*I_sc_*-plateau (Figure 2F,G).

### 3.3. The Effects of the JUUL Components on the Transepithelial Electrical Resistance

**Figure 3:**
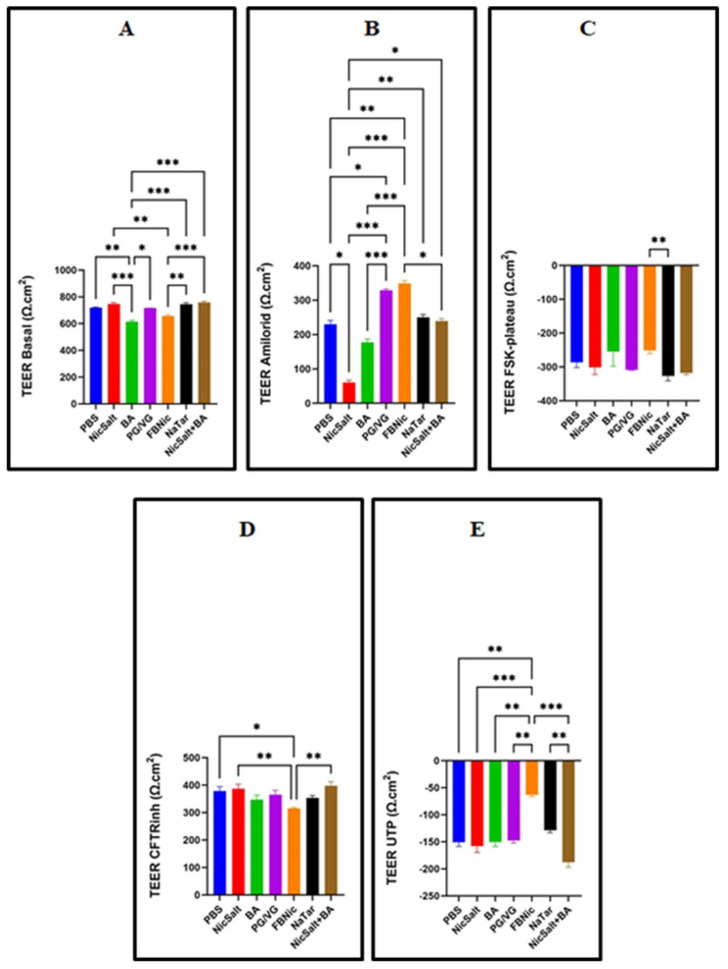
Transepithelial electrical resistance (TEER) changes in HBECs after 24 h of exposure to e-cig components or PBS. Changes in short-circuit current at baseline (**A**) and upon addition of amiloride (**B**), FSK-plateau (**C**), CFTRinh-172 (**D**), and UTP-plateau (**E**) were measured (* *p* < 0.05, ** *p* < 0.01 and *** *p* < 0.001).

TEER, a parameter measuring the electrical resistance of epithelial cell layers, is a strong indicator of cellular barrier integrity. TEER was measured in HBECs using Ussing chambers following 24 h exposure to different substances. The analysis revealed statistically significant differences among exposure groups (*p* < 0.05, Figure 3). The lowest basal TEER values were observed in BA-exposed cells (Figure 3A). After amiloride addition, a significant TEER decrease was noted in NicSalt-exposed cells (Figure 3B), while forskolin-induced CFTR activation resulted in the lowest TEER in NaTar-exposed cells (Figure 3C). Inhibition of CFTR with CFTRinh-172 led to the lowest TEER in FBNic-exposed cells (Figure 3D). Lastly, following UTP activation of CaCC channels, the lowest TEER value was observed in NicSalt+BA-exposed cells (Figure 3E).

### 3.4. Cytotoxic Effects of the JUUL Components

**Figure 4:**
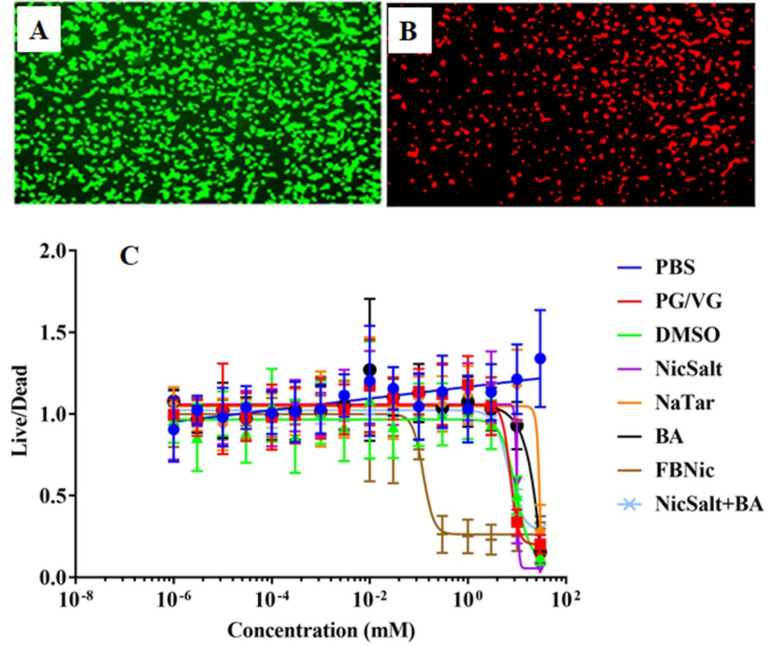
E-cig components cause cytotoxicity. HEK293T cells were incubated with different concentrations of e-liquids and stained with calcein-AM (green, live) and propidium iodide (red, dead). Representative merged images of green (calcein) and red (propidium iodide) fluorescence from live cells (**A**) and dead cells (**B**), Dose–response curve for NicSalt, BA, NaTar, PG/VG, FBNic, NicSalt+BA and DMSO (**C**).

Previous studies have demonstrated that the composition of e-liquids is highly heterogeneous, leading to varying levels of cell toxicity [23]. However, the main components of the JUUL, such as NicSalt, BA, and their combination [11], have not previously been compared in terms of cytotoxic effects with NaTar, PG/VG, or FBNic. To compare the cytotoxic effects of JUUL components (NicSalt, BA, and their combination) with NaTar, PG/VG, and FBNic, dose–response experiments were conducted on HEK293T cells (Table 2). No significant difference in cytotoxicity was observed at concentrations ranging from 999.10^−7^ mM to 0.03 mM for the substances exposed for 24 h (*p* > 0.05). When the cytotoxic effects of all substances at concentrations ranging from 0.0999 to 3 mM were compared, FBNic was found to have the most cytotoxic properties (*p* < 0.05). At concentrations ranging from 9.99 to 30 mM, all components were found to be more cytotoxic compared to the PBS group (*p* < 0.05) (Figure 4A–C).

## 4. Discussion

In recent years, the use of JUUL, a next-generation e-cigarette, has become increasingly popular, especially among young people. This trend has raised significant concerns about the potential health impacts of its use. One key area of investigation is the effect of JUUL compounds, particularly nicotine salts, on the ASL, which plays a critical role in maintaining respiratory system homeostasis.

These compounds influence the ASL height, which is regulated by ion channels such as ENaC, CFTR, and CaCC. Their impact on respiratory health is a crucial subject of study. Furthermore, assessing the cytotoxic effects of these substances is essential for a comprehensive assessment of the potential risks associated with JUUL use.

Confocal microscopy is extensively employed for measuring ASL height, enabling the study of how airway epithelia regulate ASL height [25]. In addition, the Ussing chamber provides a valuable electrophysiological method for measuring the transport of electrolytes, ions, nutrients, and drugs across epithelial tissues [26]. Together, these techniques support research demonstrating that effective mucus transport requires proper regulation of ENaC, CFTR, and CaCC activity to sustain a hydrated ASL [25].

The height and content of the ASL might be influenced by various substances that affect the ion channels involved in regulating the volume of airway surface liquid [27]. In the present study, we investigated the effects of the substances, including PBS, NicSalt, BA, PG/VG, FBNic, NaTar, and a combination of NicSalt and BA on the airway ASL height, ion transportation and TEER of HBECs. Moreover, the main components of the JUUL, Nic Salt, BA, and their combination are compared with NaTar, PG/VG, or FBNic in terms of cytotoxicity.

In the present study, our findings revealed that immediate application of JUUL components did not significantly alter ASL height initially, suggesting that HBECs may exhibit delayed or adaptive responses to these substances. However, prolonged exposure, especially to FBNic, significantly reduced ASL height, indicating cumulative effects that could impair airway mucosal integrity. A notable reduction in ASL height was observed even in HBECs cultures treated only with PBS, which served as the vehicle control in this study. This reduction may initially appear unexpected; however, it aligns with previous findings indicating that differentiated airway epithelial cell cultures naturally exhibit a gradual decrease in ASL height over time due to intrinsic physiological mechanisms, including evaporation and active absorption of the liquid layer by epithelial cells [25,26] [15,27]. Thus, the ASL height decrease observed in PBS-treated controls at 24 h and immediately after adenosine addition (AD 0 min) is consistent with the expected physiological behavior of HBECs cultures and does not reflect any specific chemical-induced response.

Consistent with our findings, a study by Woodall et al. reported that HBECs exposed to 3% 55/45 PG/VG for 90 min initially increased ASL height within the first 10 min but subsequently decreased. They suggested that this fluctuation might be attributed to the hyperosmolar properties of PG/VG or a weakening of intercellular barrier function. These observations collectively highlight the complex and dynamic nature of ASL regulation in response to e-cigarette components, emphasizing the potential long-term consequences of prolonged exposure on airway health [12].

Furthermore, despite HBECs’ ability to adapt ASL levels over time, our results indicate that NicSalt and FBNic disrupt key ion channels (ENaC, CFTR, and CaCC), with FBNic exerting a stronger effect due to its enhanced membrane permeability. Supporting our findings, previous studies have shown that exposure to e-cigarette vapor, including JUUL aerosol and e-liquid-treated neutrophil secretions, increases ENaC activity and reduces ASL height [13,14,16]. Similarly, exposure of HBECs to bronchoalveolar lavage fluid (BALF) from JUUL users enhanced ENaC activity and decreased ASL height [15]. Collectively, these results emphasize the need for further research into the long-term respiratory risks associated with JUUL use and the potential role of CFTR-targeted interventions in mitigating ASL disruption.

Identifying changes in short-circuit current and transepithelial voltages mediated by functional channels and transporters in the HBECs’ apical and basolateral membranes is crucial for maintaining airway homeostasis. Consistent with previous research [16], our findings demonstrate that e-cigarette-derived substances can significantly modulate ion channel activity, as reflected by the marked differences in Δ*I_sc_* values across exposure groups. These alterations underscore the potential disruption of airway homeostasis, highlighting the physiological consequences of prolonged exposure to these substances.

After 24 h of exposure, significant Δ*I_sc_* alterations were observed in key ion transport pathways, including ENaC, CFTR, and CaCC. NicSalt notably decreased basal Δ*I_sc_*, indicating disrupted ionic balance and ASL homeostasis. FBNic exposure further reduced Δ*I_sc_* following ENaC inhibition and CFTR blockade, suggesting enhanced CFTR suppression or altered Na^+^/Cl^−^ transport. PG/VG impaired forskolin-induced CFTR activation, while BA and FBNic significantly disrupted CaCC function.

These data indicate that, as shown in previous studies [13,15], NicSalt and FBNic affect both Na^+^ absorption [28] and Cl^−^ secretion pathways, CFTR, which are essential for maintaining proper airway homeostasis. In addition, the reduction in ∆*I_sc_* values after CFTRinh-172 exposure in FBNic-treated cells suggests enhanced CFTR inhibition or secondary disruption of chloride transport pathways. The inhibition of these pathways could potentially impair mucociliary clearance, increasing the risk of respiratory infections and other pulmonary complications [13,15].

Other studies have also shown that acute e-cig vapor causes dysfunction of CFTR and CaCCs. These deleterious effects can promote mucus thickening and decreased mucus clearance, as found in COPD patients studies [17,29,30].

This study also aimed to assess the impact of e-cigarette components on the transepithelial electrical resistance (TEER) of HBECs, a key indicator of epithelial barrier integrity. Using Ussing chambers with voltage electrodes, TEER measurements after 24 h exposure to e-cigarette substances revealed statistically significant differences among groups, highlighting their potential to disrupt epithelial function. These findings align with previous research demonstrating the effects of e-cigarette components, including JUUL, on transepithelial resistance in cell cultures [31,32]. BA exposure resulted in the lowest basal TEER, indicating increased epithelial permeability, while NicSalt significantly reduced TEER following ENaC inhibition, suggesting impaired Na^+^ transport and barrier dysfunction [31,32]. Similarly, NaTar exposure led to the lowest TEER after CFTR activation by forskolin, implying chloride transport disruption and potential imbalances in airway surface liquid hydration. FBNic exposure significantly compromised epithelial barrier integrity beyond CFTR inhibition, likely through disruptions in tight junction proteins, increased paracellular permeability, or alterations in other ion transport pathways. The substantial TEER reduction, even in the presence of CFTRinh-172, aligns with studies showing that freebase nicotine weakens epithelial resistance, induces cellular stress, and disrupts barrier function [33,34,35]. These findings highlight the detrimental impact of e-cigarette components on airway barrier function, which is critical for maintaining proper hydration and mucociliary clearance. NicSalt and BA together exhibited a synergistic weakening effect, while PG/VG exposure increased permeability and reduced TEER. Consistent with prior studies, nicotine-containing e-liquids and vapor further compromised airway epithelial integrity, underscoring the potential respiratory risks of prolonged e-cigarette exposure [12,18,36].

Given the diverse composition of e-liquid components and their widespread use, the cytotoxicity of these substances has been a subject of increasing concern. Our study builds upon previous research that has established the heterogeneous nature of e-liquid formulations especially of JUUL [37,38], and their variable impacts on cell viability [11,23]. Dose–response experiments on HEK293T cells revealed no significant cytotoxicity at lower concentrations (9.99 × 10^−7^ mM to 0.03 mM), aligning with literature suggesting minimal harm at low exposure levels [11,23,31]. However, at moderate concentrations (0.0999 mM to 3 mM), FBNic exhibited significantly higher cytotoxicity (*p* < 0.05), indicating a greater risk to cell viability than NicSalt. At higher concentrations (9.99 mM to 30 mM), all tested substances demonstrated increased cytotoxicity, reinforcing the dose-dependent nature of e-liquid toxicity [11,23]. These findings highlight the variability in e-liquid component toxicity and suggest that their combined effects warrant further investigation. Consistent with previous studies, NicSalt and BA showed no cytotoxicity when combined at low concentrations [11], whereas JUUL flavoring chemicals have been reported to exert cytotoxic effects [32].

## 5. Conclusions

In conclusion, our study demonstrated that the main chemical constituents of JUUL e-cigarettes, including nicotine salts (NicSalt), benzoic acid (BA), freebase nicotine (FBNic), sodium hydrogen tartrate (NaTar), and propylene glycol/vegetable glycerin (PG/VG), have distinct effects on airway epithelial function in HBECs cultures. Initially, acute exposure to these substances did not significantly alter airway surface liquid (ASL) height, suggesting an initial cellular adaptation or delay physiological responses. However, our findings highlight a significant health risk associated with JUUL e-cigarettes. Prolonged exposure, particularly to FBNic, led to a notable reduction in ASL height, indicating potential cumulative impairment of airway surface liquid regulation and epithelial homeostasis. This underscores the potential long-term health implications of using JUUL e-cigarettes. Further assessments revealed significant disruptions in epithelial ion transport processes, particularly involving ENaC, CFTR, and CaCC channels, as indicated by changes in transepithelial short-circuit current (Δ*I_sc_*). Additionally, our results demonstrated that both NicSalt and FBNic adversely affected the transepithelial electrical resistance (TEER), a critical indicator of epithelial barrier integrity, suggesting impaired barrier function following exposure.

Notably, the present study highlights potential limitations, including using PBS as a solvent and assessing the cytotoxicity of individual JUUL components separately without considering possible synergistic, additive, or antagonistic interactions. Future investigations should address these limitations by performing preliminary dose–response experiments specific to the tested cell type and exploring the combined toxicity of JUUL e-liquid mixtures to better mimic real-life vaping scenarios. Our findings contribute valuable mechanistic insights regarding the potential respiratory health risks of JUUL e-cigarette components. They also highlight the necessity of continued research to fully understand their biological impact on airway epithelial function and integrity.

## Figures and Tables

**Table 1:** Summary of some studies on the cellular effects of e-cigarette components on ASL height, ion transport, and epithelial barrier integrity.

Cell Types	Exposure Substance	Key Findings	References
HBECs	3% PG/VG	3% PG/VG mixture initially increased ASL height transiently, followed by a significant decrease, suggesting barrier disruption and potential cellular stress.	[12]
HBECs	E-cig aerosols	Aerosolized nicotine e-liquids significantly reduced ASL height by impairing ENaC and CFTR ion channel functions, compromising airway epithelial integrity.	[13]
HBECs	E-cig vapor	E-cig vapor significantly reduced ASL height by impairing CFTR ion channel functions and compromising airway epithelial integrity.	[14]
HBECs	Bronchoalveolar lavage fluid (BALF) from non-smokers, smokers, and vapers	JUUL e-liquids increased ENaC channel activity, reduced ASL height, and compromised airway mucociliary clearance mechanisms.	[15]
NHBECs	E-cigarette vapours or nicotine solutions	E-cigarette vapours or nicotine solutions reduced ASL height, and compromised airway mucociliary clearance mechanisms.	[16]
HBECs	50% PG/VG	PG/VG aerosols significantly decreased mucus hydration by impairing CFTR ion channel functions, compromising airway epithelial integrity.	[17]
BEC, Calu-3 cells	E-cig vapor extract	E-cig vapor extract caused toxicity in BECs and Calu-3 cells.	[18]

**Table 2:** Groups, substances, and doses of e-cig components.

	Groups	Substance	Dissolvent	Amount (Per Culture with Dex)	Dose	Duration of Exposure (h)
**The determination of the effect of e-cig components on ASL Height**	PBS	PBS	-	14 µL	-	0–24
	Nicotine Salt (NicSalt)	Nicotine Salt	PBS	14 µL	100 µM	0–24
	Freebase Nicotine (FBNic)	Freebase Nicotine	PBS	14 µL	100 µM	0–24
	Benzoic Acid (BA)	Benzoic Acid	1 M stock solution in DMSO diluted in media	14 µL	100 µM	0–24
	Sodium Hydrogen Tartrate (NaTar)	Sodium Hydrogen Tartrate	PBS	14 µL	100 µM	0–24
	PG/VG	Propylene Glycerol + Vegetable Glycerine (55/45%)	PBS	14 µL	100 µM	0–24
	Nicotine Salt +Benzoic Acid (NicSalt+BA)	Nicotine Salt+ Benzoic Acid Mix	PBS	14 µL	100 µM	0–24
**The determination of the effects of e-cig on ion transport and TEER**	PBS	PBS	-	20 µL	-	24
	Nicotine Salt (NicSalt)	Nicotine Salt	PBS	20 µL	100 µM	24
	Freebase Nicotine (FBNic)	Freebase Nicotine	PBS	20 µL	100 µM	24
	Benzoic Acid (BA)	Benzoic Acid	1 M stock solution in DMSO diluted in media	20 µL	100 µM	24
	Sodium Hydrogen Tartrate (NaTar)	Sodium Hydrogen Tartrate	PBS	20 µL	100 µM	24
	PG/VG	Propylene Glycerol + Vegetable Glycerine (55/45%)	PBS	20 µL	100 µM	24
	Nicotine Salt +Benzoic Acid (NicSalt+BA)	Nicotine Salt+ Benzoic Acid Mix	PBS	20 µL	100 µM	24
**The determination of cytotoxic effects of E-Cig**	PBS	PBS	-	20 µL	999.10^−7^–30 mM	24
	Nicotine Salt (NicSalt)	Nicotine Salt	PBS	20 µL	999.10^−7^–30 mM	24
	Freebase Nicotine (FBNic)	Freebase Nicotine	PBS	20 µL	999.10^−7^–30 mM	24
	Benzoic Acid (BA)	Benzoic Acid	1 M stock solution in DMSO Diluted in Media	20 µL	999.10^−7^–30 mM	24
	Sodium Hydrogen Tartrate (NaTar)	Sodium Hydrogen Tartrate	PBS	20 µL	999.10^−7^–30 mM	24
	PG/VG	Propylene Glycerol + Vegetable Glycerine (55/45%)	PBS	20 µL	999.10^−7^–30 mM	24
	DMSO	Dimethyl Sulfoxide	-	20 µL	999.10^−7^–30 mM	24
	Nicotine Salt + Benzoic Acid (NicSalt+BA)	Nicotine Salt+ Benzoic Acid Mix	PBS	20 µL	999.10^−7^–30 mM	24

## Data Availability

The original data are available to researchers upon request.
